# Global Repetition Influences Contextual Cueing

**DOI:** 10.3389/fpsyg.2018.00402

**Published:** 2018-03-27

**Authors:** Xuelian Zang, Artyom Zinchenko, Lina Jia, Leonardo Assumpção, Hong Li

**Affiliations:** ^1^Key Research Base of Humanities and Social Sciences in the Ministry of Education, Academy of Psychology and Behavior, Tianjin Normal University, Tianjin, China; ^2^College of Psychology and Sociology, Shenzhen Key Laboratory of Affective and Social Cognitive Science, Shenzhen University, Shenzhen, China; ^3^Experimental Psychology, Department of Psychology, Ludwig-Maximilians-Universität München, Munich, Germany; ^4^Department of Education, School of Humanities, Jiangnan University, Wuxi, China; ^5^Center for Neuroimaging, Shenzhen Institute of Neuroscience, Shenzhen, China

**Keywords:** contextual cueing, global repetition, implicit learning, contextual transfer, presentation ratio

## Abstract

Our visual system has a striking ability to improve visual search based on the learning of repeated ambient regularities, an effect named contextual cueing. Whereas most of the previous studies investigated contextual cueing effect with the same number of repeated and non-repeated search displays per block, the current study focused on whether a global repetition frequency formed by different presentation ratios between the repeated and non-repeated configurations influence contextual cueing effect. Specifically, the number of repeated and non-repeated displays presented in each block was manipulated: 12:12, 20:4, 4:20, and 4:4 in Experiments 1–4, respectively. The results revealed a significant contextual cueing effect when the global repetition frequency is high (≥1:1 ratio) in Experiments 1, 2, and 4, given that processing of repeated displays was expedited relative to non-repeated displays. Nevertheless, the contextual cueing effect reduced to a non-significant level when the repetition frequency reduced to 4:20 in Experiment 3. These results suggested that the presentation frequency of repeated relative to the non-repeated displays could influence the strength of contextual cueing. In other words, global repetition statistics could be a crucial factor to mediate contextual cueing effect.

## Introduction

Stable memory representations of past events are essential for acquisition and integration of new information. For instance, previously relevant locations of search objects (e.g., an item on a supermarket shelf) receive attentional priority in subsequent encounters (e.g., Torralba, 2003; Brockmole et al., 2006). Such a memory acquisition can be either explicit and hypothesis driven (see Vadillo et al., 2016 for a meta-analysis study), when people actively learn new information; or implicit and without conscious awareness, when information is learned in an incidental manner (Reber, 1989; Parkin et al., 1990; Dienes and Berry, 1997). In other words, both implicit and explicit knowledge could be used to facilitate visual processing. A typical facilitation effect in visual processing is the contextual cueing effect that was first reported by Chun and Jiang (1998). In a standard contextual cueing experiment, participants search for a ‘T’ like target among ‘L’ like distractors. Unbeknownst to participants, half of the displays are repeated configurations (i.e., locations of ‘T’ and ‘L’s are maintained constant), while the other half of displays are non-repeated configurations with variable and unpredictable distractor locations. A typical response time facilitation (i.e., termed contextual cueing effect) is observed for the repeated relative to the non-repeated configurations (Chun and Jiang, 1998, 2003; Goujon et al., 2015), suggesting that the human visual search efficiency is shaped by acquisition of constant regularities in visual environment. Following the visual search task, participants usually perform an explicit recognition test during which they have to discriminate between repeated and the non-repeated configurations. A common finding is that the mean hit rates of the recognition performance was numerically (but not significantly) higher than the mean false alarm (e.g., Conci et al., 2011, 2013). A prevalent interpretation to this result pattern is that contextual cueing is mainly driven by an *implicit* memory of invariant spatial configurations (see a review, Goujon et al., 2015). Nevertheless, this interpretation was challenged by Vadillo et al. (2016), who claimed that the implicit nature of contextual learning is a false negative conclusion caused by underpowered samples of the recognition test in previous studies, given that small sample size (usually below 20) and small number of trials (usually below 40) were used. However, regardless of the explicit or implicit nature of the contextual cueing, this type of cueing is a ubiquitous effect that was repeatedly observed in previous studies (see a review Goujon et al., 2015).

A number of studies has shown that contextual cueing can be established based on different types of the statistical regularities, such as constant spatial locations among target and distractors (Brady and Chun, 2007), distractor-distractor associations (i.e., the locations of the distractors but not that of the target maintained constant, e.g., Beesley et al., 2015), repeated feature (e.g., item shapes) co-variations among target and distractors (i.e., object-based context, Chun and Jiang, 1999). In addition to these spatial- or object-based regularities, other forms of global statistics, such as how often target appear in a particular location (i.e., the probability of target location) in a display, could also be learned and engender probability cueing of the target location. For instance, in the study of Jiang et al. (2014), the target appears more often in a particular quadrant (i.e., a rich quadrant of 50% probability of containing the target) than other sparse quadrants with target presentation probability of only 16.7%. The authors observed significant probability-based contextual cueing, given that the RTs of the visual display with target in the rich quadrant were significantly faster than that in the sparse quadrant. A recent study by Tseng et al. (2011) further found that robust contextual cueing effect was modified by different types of the global statistics, namely repetition frequency of single displays. In that study, participants performed 25-blocks of visual search, with each block containing 28 repeated and 28 non-repeated configurations. For each presentation of the repeated configuration, the locations of the target and distractors maintained constant while for each presentation of non-repeated configurations, only the location of the target (but not of the distractors) was kept constant. Importantly, each configuration type (repeated and non-repeated) consisted of four configurations that were presented three times per block, four configurations that were presented two times per block and eight were presented only once per block. The results showed stronger contextual cueing effect for displays that were repeated more often (i.e., two or three times per block) relative to rarely repeated displays (once per block).

Whereas Jiang et al. (2014) examined probability-based contextual cueing, and Tseng et al. (2011) tested how contextual cueing is affected by the frequency of a single display repetition, a question remains whether other forms of global statistics may also influence contextual cueing. Specifically, we were interested in whether increasing or decreasing the number of the repeated displays relative to the number of the non-repeated displays in a given search task, but presenting each display only once per block (for an ease of argumentation, we hereafter name this manipulation as ‘global repetition’) would modulate contextual cueing. Global repetition is one of the crucial factors to form expectations about the re-occurrence of subsequent events. For instance, when individuals (explicitly or implicitly) notice multiple displays to be repeated in the first several blocks of a visual search task, they may expect the same repetition to occur in the following search blocks. On the contrary, if they encountered no repetition (or very few repetitions) at the beginning, participants may also expect no repetitions in following search blocks. Since expectation influence perception and visual search efficiency (Puri and Wojciulik, 2008; Summerfield and Egner, 2009; Jones and Kaschak, 2012), it is possible that the global repetition may influence contextual learning through modifications of expectations. Note that expectations could be formed either explicitly based on explicit cues and instructions (Eimer, 1998) or implicitly through the learning of bottom-up features ( such as probability of target’s location in Shen and Alain, 2012).

Consequently, the present study with four experiments aimed to investigate whether global repetition formed by the presentation ratio of repeated relative to non-repeated contexts mediates contextual cueing effect. In general, there are three possible methods for manipulating global repetitions in visual search: (1) reducing or increasing the number of repeated (but not non-repeated) displays, (2) changing the number of non-repeated (but not repeated) displays (e.g., Zinchenko et al., in press) or (3) reducing (or increasing) the number of repeated displays while at the same time increasing (or reducing) non-repeated displays, in order to maintain the total number of displays per block. The former two methods change the total number of trials in an experiment which affect overall experimental time and may require different amount of cognitive resources to complete the whole experiment. For instance, participants have to concentrate on the search task for longer time and devote overall more cognitive effort when the experiment contains more search trials. As a result, these changes could potentially influence contextual cueing effect. To avoid such task differences as a function of global repetition manipulation, in Experiments 1–3 we kept the total number of trials per block constant but manipulated the presentation ratio of repeated to non-repeated displays to obtain different levels of global repetition (see **Table 1** and also the following methods part). A further Experiment 4 with different total number of trials was also designed as a comparison experiment.

**Table 1:** Schematic illustrations of global repetitions and display configurations in each experiment.

Experiments	Global repetition (repeated: non-repeated)	Total number of trials	Experimental duration	Number of trials per block	Number of repeated displays per block	Number of non-repeated displays per block
1	Medium (1:1)	720	∼60 min	24	12	12
2	High (5:1)	720	∼60 min	24	20	4
3	Low (1:5)	720	∼60 min	24	4	20
4	Medium (1:1)	240	∼20 min	8	4	4

Based on the previous literature we formulated two alternative hypotheses: (1) Studies found that contextual learning is an implicit process (although see Vadillo et al., 2016 for an alternative view) that occurs automatically and without conscious awareness (Goujon et al., 2015), that is, participants do not deliberately initiate or end the learning process. Therefore it is possible that contextual learning could happen all the time irrespective of global repetitions (because participants are not able to ‘switch off’ this implicit learning process) and lead to a comparable magnitude of cueing effect among different global repetition conditions; (2) Since different global repetition statistics may change participants’ expectations regarding repetition of the following search displays, contextual learning could be modified. Specifically, when the global repetition is low, participants may expect no repetitions in the search task. In this case, they would give up the contextual learning process to save cognitive resource.

## Experiment 1

### Participants

Because only four non-repeated configurations were used in Experiments 3 and 4 (see following), we doubled the sample size as compared to previous studies (e.g., Chun and Jiang, 1998; Zellin et al., 2014) in order to make up for the higher intra-individual variance. A total of 28 participants (mean age = 21.14 years, females = 21) took part in Experiment 1. All of them had normal or corrected-to-normal visual acuity, and were naive to the purpose of the experiment. The study was approved by the ethics committee of the Shenzhen University in China, and participants signed written consent form prior to the experiment.

### Apparatus and Stimuli

Experiment 1 was carried out in a sound attenuated, lamp illuminated booth. The viewing distance was fixed to 57 cm by having observers sitting on a chair positioned at a fixed location in front of the computer. Visual stimuli presentation was developed and controlled using Psychtoolbox (Brainard, 1997; Pelli, 1997) and Matlab codes.

**FIGURE 1:**
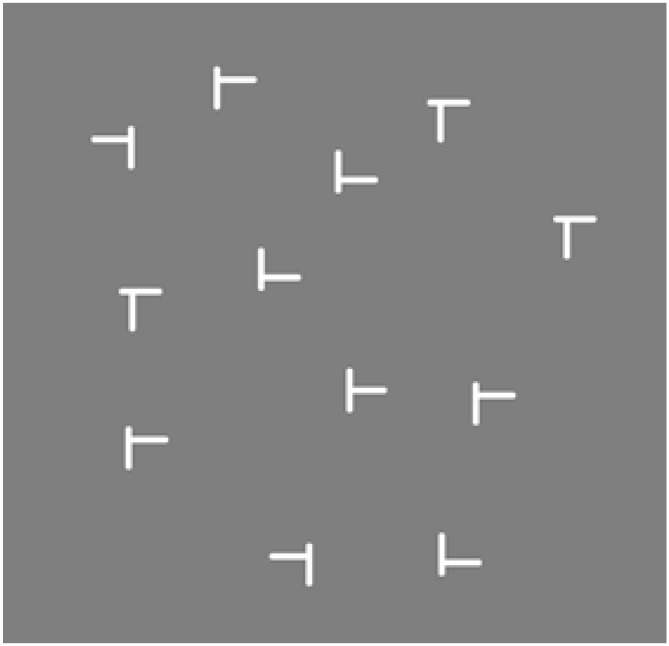
Example of the visual stimlus in the experiments.

The white visual search items (each of 0.9° × 0.9° visual angle in size, RGB value = [255 255 255]) consisting of one ‘T’-shaped target and eleven ‘L’-shaped distractors, were presented on a gray background (RGB values = [128 128 128]). Similar to previous studies (e.g., Jiang and Chun, 2001; Olson and Chun, 2002; Zang et al., 2015), the ‘L’ distractors had a small offset (0.18°) at the line junctions to make them more similar to the target ‘T.’ The ‘T’ target was rotated 90° either clockwise or counter-clockwise, pointing to the right or to the left (requiring a ‘left’ or ‘right’ response, respectively), while the ‘L’ distractors were randomly rotated 0, 90, 180, or 270° from the vertical midline. Both ‘T’ and ‘L’s were randomly placed at 64 possible locations inside an invisible 8 × 8 grid square area, with each location subtending 1.8° × 1.8° of visual angle, see **Figure 1** as example.

### Procedure and Design

Experiment 1 adopted the standard contextual cueing paradigm as a baseline experiment in the current study which consists of a 30-blocks search session and a 1-block recognition session. Each block contains 12 repeated and 12 non-repeated search trials. For the repeated display, both locations and orientations of the ‘L’ distractors, and the location of the target were kept constant and repeated once per block, while for the non-repeated display, distractors’ locations and orientations (but not target location) changed randomly in each presentation. To avoid potential response learning effect, the target orientations for both repeated and non-repeated displays were randomly assigned to the left or the right across the whole experiment.

During the search session, participants had to identify the orientation of the target letter ‘T’ as fast and accurately as possible by pressing either the left or the right arrow key on the keyboard, using their index fingers. Each trial started with the presentation of a central fixation cross that remained visible for 800–1000 ms. The offset of the fixation cross was immediately followed by a search display that was presented on the screen until a response was made, or until 10 s (in the absence of a response) had elapsed. The next trial started automatically after a random inter-trial interval of 1000–1200 ms. Participant’s correct response rate was presented on the screen at the end of each block.

During the following recognition session, the original repeated displays from the training session and a set of newly generated non-repeated configurations were presented. By pressing the left/right arrow keys (indicating yes/no response, respectively), participants made a forced choice as to whether a given display was a repeated or a non-repeated display. The displays were presented on the screen until a response was made or else for a maximum of 20 s. Response feedback was not provided.

Prior to the experiment, participants were given a practice block of 24 search trials (with random item layouts) to become familiar with the task. No configuration presented during the practice session was reused in the subsequent experiment. Participants were asked to aim for a performance level of at least 85% correct responses before the start of the main experiment. If the error rate was too high, participants were given an extra practice block (all the participants were able to continue the experiment within a maximum of two practice blocks).

### Results

In cases in which the sphericity assumption was violated, the Greenhouse–Geisser correction was applied. To increase the power of statistical analysis, every six successive search blocks were grouped together into one epoch, yielding epoch 1–5 of the search task.

All the participants finished the search task with very high accuracy performance (>99%), and the mean error rate (0.83%) was too low for reliable statistical analysis (similar results was also observed in the following experiments). In addition, RTs outside the range of 200 ms to 2.5 standard deviations of the mean of individual RTs were excluded from main RT analysis, showing discard rates of 3.15%.

#### Search Task

**FIGURE 2:**
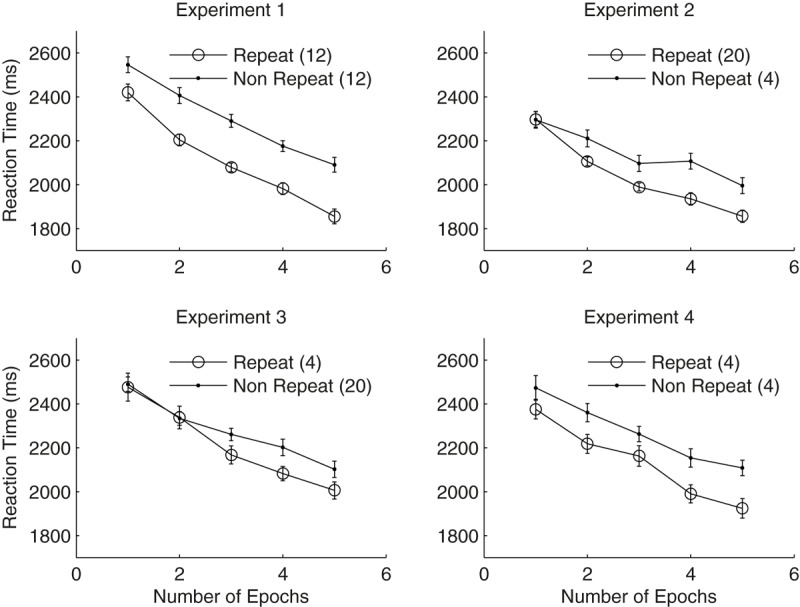
Search Performances as a function of epochs in three experiments. Lines with circles depict the mean RTs for repeated displays, whereas the lines with dots denote non-repeated displays.

Participants’ mean reaction times (RTs) of the search task were analysed by a 2 × 5 repeated-measures ANOVA with factors context (repeated vs. non-repeated) and epoch (1–5). As a result, we observed a significant main effect of both context and epoch: context, *F*(1,27) = 51.05, *p* < 0.001, $\eta_{p}^{2}$ = 0.65, mean RTs were 194 ms faster for the repeated than the non-repeated context (**Figure 2**); epoch, *F*(2.27,61.18) = 54.86, *p* < 0.001, $\eta_{p}^{2}$ = 0.67, mean RTs were 510 ms faster in epoch 5 compared to epoch 1. The context x epoch interactions didn’t reach the significant level, *F*(4,108) = 1.63, *p* = 0.17, $\eta_{p}^{2}$ = 0.057.

As shown in **Figure 2**, contextual cueing effect reached the significant level already in the first epoch [*t*(27) = 3.52, *p* = 0.002]. Further block-wise analysis (blocks 1–6) of the RTs in the first epoch showed that RT of the repeated displays was even 8.8 ms slower than that of the non-repeated displays (i.e., negative cueing effect) in the first block when participants viewed search displays for the first time, but became faster for the repeated than the non-repeated displays from block 2 onwards (mean difference of 243.3, 101.8, 185.5, 91.4, and 122.1 ms in blocks 2–6, respectively). The 2 (context) × 2 (block) repeated measures ANOVA of participants’ RT performance (in blocks 1 and 2) showed significant context × block interaction [*F*(1,27) = 5.33, *p* = 0.029, $\eta_{p}^{2}$ = 0.17]. All in all, these results suggest that a robust contextual cueing was established in the current experiment, and it had a quick onset after the start of the experiment (i.e., from block 2 onwards). Comparable rapid contextual learning effect was also observed in previous studies (Conci and von Mühlenen, 2009, 2011).

#### Recognition Test

The recognition data of one participant was excluded for the analysis as s/he pressed the same response key during the entire recognition test (s/he considered all the recognition displays as repeated displays), yielding 100% of the mean hit (i.e., correctly identified repeated displays) and false alarm rate (i.e., reported the non-repeated display as repeated one). All the other participants were able to respond to the recognition trials within the time limitation (20 s). The overall mean hit rate (57.41%) was numerically higher than the mean false alarm rate (53.70%), but the recognition sensitivity (*d*′ prime) was not different from 0, *t*(26) = 1.29, *p* = 0.21. Because previous studies hold controversial opinions on the implicit and explicit nature of contextual cueing (Chun and Jiang, 1998; Vadillo et al., 2016), here we choose to not draw any strong conclusion on the recognition test given that only 24 trials were involved in the recognition test.

### Discussion

Experiment 1 was designed as a baseline condition to investigate whether global repetition frequency of 1:1 could elicit a reliable contextual cueing effect. Specifically, standard contextual cueing paradigm with 12 repeated and 12 non-repeated search configurations (i.e., global repetition = 1:1) was used during the experiment, and similar results were observed, that is robust contextual cueing effect was established during the search session. RTs of the repeated displays were significantly faster than that of the non-repeated displays after contextual learning (similar results see the seminar study of Chun and Jiang, 1998).

## Experiment 2

Experiment 2 was designed to investigate whether a higher ratio of global repetition results in stronger contextual cueing effect. The experimental paradigm was essentially the same as in Experiment 1 except each experimental block contained 20 repeated and 4 non-repeated configurations, leading to a global repetition of 5:1. In addition, a different group of 28 participants (20 females, mean age = 20.89 years) took part in the experiment.

### Results and Discussion

Similar data analysis as in Experiment 1 was applied in the current Experiment 2. Overall mean error rates and discard rates were low (0.76% and 3.09%, respectively).

#### Search Task

Repeated-measures ANOVA with context and epoch as factors revealed significant main effects of both context and epoch: context, *F*(1,27) = 15.19, *p* < 0.001, $\eta_{p}^{2}$ = 0.36, with mean contextual cueing effect of 104 ms; epoch, *F*(2.8,75.68) = 31.81, *p* < 0.001, $\eta_{p}^{2}$ = 0.54, RTs in epoch 5 was 337 ms faster than that in epoch 1. The context x epoch interaction was also significant, *F*(4,108) = 2.48, *p* = 0.048, $\eta_{p}^{2}$ = 0.08, mainly caused by the non-significant contextual cueing effect in epoch 1 [*t*(27) = 0.059, *p* = 0.95], but significant cueing effect from epoch 2 onwards (all *t*s > 2.14 and all *p*s < 0.041). Taken together, these results suggest both contextual cueing and procedural learning effect was manifested when the global repetition was high (5:1, with 20 repeated and 4 non-repeated displays per block).

Note participants’ overall mean RTs were 2034.5 and 2138.8 ms of the repeated and non-repeated contexts, respectively, which were numerically faster than the RTs in Experiment 1 (2104.9 and 2297.8 ms of the repeated and non-repeated contexts, respectively), but this trend didn’t reach significant level (all *F*s < 2.04, *p*s > 0.05). In order to compare the strength of contextual cueing effect between the global repetitions of 1:1 (i.e., Experiment 1) and 5:1 (i.e., Experiment 2), mean contextual cueing effect in the last search epoch (when contextual cueing effect reached the peak level) were subjected to one-way between groups ANOVA with experiment as factor. The results revealed non-significant between group (i.e., experiments) effect, *F*(1,54) = 2.03, *p* = 0.160, although the mean contextual cueing effect was numerically smaller than that in Experiment 1 (139.6 ms vs. 235 ms).

These results suggest that the well-established contextual cueing effect (i.e., after four epochs of learning) was comparable between Experiment 1 (with global repetition of 1:1) and Experiment 2 (with global repetition of 5:1). In other words, increasing the global repetition from 1:1 to 1:5 cannot increase general contextual cueing effect, which probably due to a ceiling effect of contextual learning.

#### Recognition

The mean hit rate was 61.61%, which was significantly higher than the false alarm rate 48.21% given that the recognition sensitivity (*d*′ prime) was significantly higher than 0 [*t*(27) = 2.83, *p* = 0.009]. These results showed strong evidence for the alternative hypothesis, that is explicit contextual memory exist in the current study.

## Experiment 3

Experiment 3 aimed to investigate whether a low global repetition frequency (1:5) constraints contextual learning. The experimental paradigm is essentially the same as previous two experiments except each experimental block contains 4 repeated and 20 non-repeated configurations. Another different group of 28 participants (16 females, mean age of 21.82 years) took part in the experiment.

### Results and Discussion

Similar to previous experiments, participants’ overall mean error rates and discard rates were low: 0.71 and 3.01%, respectively.

#### Search Task

Repeated-measures ANOVA with context and epoch as factors revealed significant main effects of epoch [*F*(2.52,68.07) = 29.08, *p* < 0.001, $\eta_{p}^{2}$ = 0.52, RTs in epoch 5 was 429 ms faster than in epoch 1], but not of context and context × epoch interaction [context, *F*(1,27) = 1.87, *p* = 0.18, $\eta_{p}^{2}$ = 0.065, although mean RTs of the repeated context was 64 ms faster than that of the non-repeated context; context × epoch, *F*(4,108) = 1.79, *p* = 0.14, $\eta_{p}^{2}$ = 0.06], suggesting no significant contextual cueing effect was observed in the current Experiment 3 when the global repetition is low (1:5).

Note RTs of repeated displays were numerically faster than that of non-repeated displays from epoch 3 onwards: the mean difference of 93.3 ms [*t*(27) = 1.62, *p* = 0.12], 119.6 ms [*t*(27) = 2.49, *p* = 0.019] and 96.3 ms [*t*(27) = 1.82, *p* = 0.08], respectively, none of them reached statistical significant level according to epoch-wise *t*-test with Bonferroni correction (criterial of 0.01). When comparing the numerical cueing effect in the last epoch (i.e., epoch 5) to our baseline condition (epoch 5 in Experiment 1), one-way between groups ANOVA with experiment as factor showed significant between group effect, *F*(1,54) = 4.23, *p* = 0.045, suggesting the numerical cueing effect in the current experiment was significantly weaker than the cueing effect (235 ms) in Experiment 1 (global repetition = 1:1). These results suggest that participants’ contextual learning ability was greatly reduced (to a non-significant level) within 5 epochs of training when the global repetition was low (1:5). Of note, it is still possible that a significant contextual cueing effect would have been developed if we had presented more training epochs in the current Experiment 3 (for example 10 rather than 5). However, it is clear that participants’ learning ability (at least within 5 epochs of training) was limited under the low global repetition condition, as contextual facilitation was much weaker than the baseline experiment.

To further compare participants response behavior among Experiments 1–3, participants’ overall RT performance were subject to 2 (context: repeated vs. non-repeated) × 5 (epoch 1–5) repeated measures ANOVA with Experiments (1–3) as between group factor. The results showed significant main effect of context [*F*(1,81) = 36.45, *p* < 0.001], epoch [*F*(4,324) = 110.95, *p* < 0.001], but not of experiments [*F*(1,81) = 1.73, *p* = 0.18]. The two-way interactions of context × epoch and context × experiments reached significant level: context × epoch: *F*(4,324) = 4.69, *p* < 0.01, suggesting contextual cueing developed by training; context × experiments: *F*(2,81) = 3.67, *p* = 0.03, which is caused by significant main effect of context in Experiments 1 and 2 but not 3 (see results above); No other interactions reached significant level (all *p*s > 0.29). These results further confirmed our hypothesis, that low global repetition may impede participants’ contextual learning ability.

#### Recognition

Participants failed to respond to 0.30% of the recognition trials within the time limitation (20 s), and these trials were removed in the follow up analysis. The mean hit rate was 58.93%, and was quite similar to the mean false alarm rate of 57.50%. The recognition sensitivity (*d*′ prime) was not different from 0, *t*(26) = 0.96, *p* = 0.35, consistent with the finding of no contextual cueing effect during the search task in the current experiment.

## Experiment 4

It is important to note that the number of repeated trials changed from 360 to 600, and to 120 in Experiments 1–3, respectively, this different number of repeated configurations per experiment may also influence contextual cueing. To exclude this potential confounding, Experiment 4 with 240 trials consisting of 120 repeated (similar to Experiment 3, each block contained four repeated configurations) and 120 non-repeated trials was adopted to examine whether the absolute number of repeated configurations could additionally modulate contextual cueing. The global repetition was set as 1:1 (similar to Experiment 1). Another 28 participants (16 females, mean age of 21.96) took part in the experiment. The experimental design and paradigm, in all the other aspects, were the same as previous experiments.

### Results and Discussion

#### Search Task

Participants’ overall mean error and discard rates were 1.32 and 3.74%, respectively. Repeated-measures ANOVA of mean RT with context and epoch as factors revealed significant main effects of both epoch and context: epoch, *F*(2.91,78.63) = 24.53, *p* < 0.001, $\eta_{p}^{2}$ = 0.48, RTs in epoch 5 was 409 ms faster than in epoch 1; context, *F*(1,27) = 12.83, *p* < 0.001, $\eta_{p}^{2}$ = 0.32, with mean contextual cueing effect of 138 ms. The context x epoch interaction didn’t reach the significant level [*F*(4,108) = 0.48, *p* = 0.75, $\eta_{p}^{2}$ = 0.02], suggest an early onset of (numerical) contextual cueing effect in the current experiment, although the cueing effect in epoch 1 (97 ms) didn’t reach significance [*t*(27) = 1.57, *p* = 0.13].

When comparing the significant contextual cueing facilitation in the last epoch between Experiment 1 (i.e., baseline condition that contained overall 720 trials) and Experiment 4 (contained overall 240 trials), one-way between groups ANOVA with experiment as factor showed non-significant between-group effect, *F*(1,54) = 0.51, *p* = 0.48, suggesting the amount of contextual cueing effect (235 ms vs. 184.7 ms) was comparable between the two experiments. Further repeated measures ANOVA with context (repeated vs. non-repeated) and epoch (1–5) as within subject factor, Experiments (1 and 4) as between subject factor revealed significant main effect of context [*F*(1,54) = 49.66, *p* < 0.001] and epoch [*F*(4,216) = 72.04, *p* < 0.001]. No other effects reached significant level (all *p*s > 0.24). These results further confirmed comparable contextual cueing behavior between current Experiment 4 and the baseline Experiment 1. In other words, merely changing the overall number of trials of the repeated displays (from 12 in Experiments 1–4 in Experiment 4) but maintained global repetition (1:1) does not impede contextual cueing effect.

#### Recognition

We observed no significant differences between the mean hit rate (55.77%) and the mean false alarm rate (52.88%): the recognition sensitivity (*d*′ prime) was not different from 0, *t*(27) = 0.52, *p* = 0.61.

To further increase the statistical power of recognition data analysis, we collapsed participants’ recognition performance in Experiments 1, 2, and 4 (i.e., the experiments that exhibited significant contextual cueing effect). This resulted in 83 participants [one participant in Experiment 1 was excluded because s/he responded positively (e.g., ‘yes’) during the whole recognition phase] and 1992 recognition trials. This whole data set was subject to one-way repeated measures ANOVA, with the results showed overall higher the mean hit rates (58.4%) than the false alarm rates (50.3%). The recognition sensitivity (*d′* prime) was marginal significantly larger than 0, *t*(27) = 1.79, *p* = 0.08, suggesting some weak evidence on contextual learning to be explicit. These results support previous meta-analysis study by Vadillo et al. (2016), that challenged the common belief of implicit nature of contextual learning. Vadillo et al. (2016) concluded that recognition tests in previous studies lacked statistical power, and evidence on explicit memory of contextual cueing could be observed when the recognition task contained either more participants or more trials. In the current study, we found no significant recognition sensitivity in Experiments 1 and 4, but marginal significant evidence of explicit memory (at least to some extent) in contextual cueing when collapsing all the experiments and significant recognition sensitivity in Experiment 2. Note the effect size of the collapsed recognition test is still low ($\eta_{p}^{2}$ = 0.006). Therefore, based on these recognition analyses, we believe some explicit memory in contextual cueing may exist.

Finally, because our main focus in the current study is contextual cueing, we further compared the strength of contextual cueing of the displays that were explicitly memorized (i.e., hit displays in the recognition test) to the missed repeated displays that were recognized as non-repeated. Specifically, the search trials of the last training epoch (i.e., epoch 5) in all the four experiments were classified into three groups according to participants’ recognition performance: non-repeated displays (6578 trials), correctly recognized repeated displays (3939 trials) and non-recognized repeated displays (2645 trials). The mean of the non-repeated trials of each participant were served as baseline to calculate contextual cueing effect of the recognized displays (RT_non-repeated_ – RT_recognized_) and the non-recognized repeated displays (RT_non-repeated_ – RT_non-recognized_). Data of all the four experiments were collapsed together to increase the statistical power and Pearson correlation analysis with individual configurations as observations revealed no significant correlation between the amount of contextual cueing and the recognized/non-recognized displays (*r* = -0.005, *p* = 0.66). Further One-way ANOVA analysis of mean contextual cueing effect also revealed no significant group difference between the recognized (mean contextual cueing of 140.7 ms) and non-recognized (mean cueing of 192.7 ms) displays: *F*(1,100) = 1.50, *p* = 0.223, $\eta_{p}^{2}$ = 0.015. These results suggest no significant cueing difference between the displays that were explicitly recognized and the displays that were not recognized (similar findings see also Colagiuri and Livesey, 2016).

## General Discussion

The presented study with four experiments investigated whether global repetition frequency influences contextual learning. Specifically, in the first three experiments, we manipulated the presentation ratio between repeated and non-repeated displays per experimental block: 12 repeated and 12 non-repeated displays in the baseline Experiment 1 (global repetition = 1:1); 20 repeated vs. 4 non-repeated displays in Experiment 2 (global repetition = 5:1), and 4 repeated vs. 20 non-repeated displays in Experiment 3 (global repetition = 1:5). The results showed significant contextual cueing effect in both Experiments 1, 2, and 4 when the global repetition frequency was high (≥ 1:1 ratio). Note the amount of contextual cueing effect between Experiments 1 and 2 were comparable which could be caused by the ceiling effect: when contextual learning reached its maximum magnitude already in the global repetition of 1:1 (in Experiment 1), it could not be further increased in Experiment 2 with global repetition of 5:1. Importantly, Experiment 3 (with low global repetition of 1:5) observed no significant cueing effect. Although there was a numerical RT trend toward facilitation (see **Figure 2**) in the later stage of the Experiment (from epoch 4 onwards), this trend did not reach significance. Further this numerical RT facilitation of the repeated context in the last search epoch was significantly smaller than that in Experiment 1, suggesting low global repetition (1:5) hinders contextual learning. Note similar findings were also observed in our recent study (Zinchenko et al., in press) that tested different presentation ratios of repeated displays (20%, 50% vs. 80%) within the same group of participants. That is participants showed significant contextual cueing effect with repetition ratio of 50 and 80% but not of 20%.

Note that together with the changes of the global repetition across Experiments 1–3, the number of repeated trials (i.e., 20, 12, and 4, respectively) per block also changed which could be a potential confounding factor in Experiment 3 (when global repetition is low). However, this explanation is less likely. First of all, a recent study by Assumpção et al. (2015) observed a robust contextual cueing effect in a tactile search task even when only four repeated and four non-repeated configurations per block were presented. In addition, Schlagbauer et al. (2012) showed that the overall cueing effect observed in a contextual-guided visual search task, derived from 3 to 4 (out of 12) learned repeated displays. To further exclude this possible explanation, we designed Experiment 4 as a control experiment, which contained the same number of repeated configurations as in Experiment 3 (i.e., 4) while preserving the same global repetitions as in Experiment 1 (1:1). With this manipulation, we again observed robust contextual cueing effect within five epochs of training, and the amount of contextual cueing effect was comparable to the baseline Experiment 1 (12 repeated vs. 12 non-repeated displays), suggesting that four repeated configurations could offer enough power to ensure contextual cueing. Hence the lack of contextual cueing effect in Experiment 3 was caused by the low presentation ratio of the repeated configurations rather than the insufficient number of these configurations. Taken together, the results of the four experiments showed that irrespective of the number of trials per block, the contextual cueing effect manifests when the global repetition is at a medium or high level (≥1:1) but not when it is low (1:5).

The finding that presentation rates of repeated context influence contextual cueing was also supported by a previous study from Yang and Merrill (2015). The authors found that contextual cueing in children was influenced by the presentation frequency of repeated displays, as significant contextual cueing effect was established following high (i.e., 100% or 67%) but not low (i.e., 33%) presentation frequency of the repeated contexts. Remarkably, with the same experimental design, young adults (≥18 years old) revealed robust contextual cueing in all the three conditions. The authors claimed that the impact of the presentation frequency of repeated displays in contextual cueing could due to the cognitive development states of children and adults. That is, contextual learning in young childhood may be more easily disrupted than that of adults by the presentation of novel (or noisy) displays. Importantly, Yang and Merrill (2015) adjusted the difficulty of the visual search task in their experiments for the children by using cartoons as the search items. This type of search task, however, might have been too easy for young adults to perform. As a result, the authors observed no impairment in adults’ contextual learning even when repeated context was presented on 33% of the trials. In contrast to Yang and Merrill (2015), the current study demonstrated that, at least with the standard contextual cueing search task (with ‘T’ and ‘L’s as search items), adults’ contextual learning ability is susceptible to the lower presentation frequency of the repeated relative to the non-repeated contexts (i.e., Experiment 3: 1:5).

Given that the global repetition represents a type of temporal feature (that could be learned through multiple blocks of the observations of the search trials), the present study shows that this feature can play an important role in the spatial contextual learning, especially when the global repetition is rare. Note also that the learning of global repetition could modify one’s expectations of the upcoming information: participants may (temporally) switch on (or off)^1^ the cueing learning process by acquiring adequate (or inadequate) statistical regularities in the present study (namely global repetition). In other words, when repeated configurations are presented with higher frequency, participants could form a strong expectation on the repetitions of the upcoming stimuli and ‘switch on’ contextual learning process that further speeding up visual search to repeated contexts (although recruiting more cognitive resources). However, when the global repetition frequency is too rare, contextual memory representation is poor and the visual system may expect no (or very infrequent) repetitions on the upcoming stimuli, hence ‘switch off’ the context learning process in order to save cognitive resources. Note that the evidence on whether repeated stimuli exist and how often do these stimuli repeat can be updated with the progress of the experiment. That is, the evidence increases with the occurrence of the repeated stimuli and decreases with continuous presentation of novel stimuli. Therefore, although the numerical RT facilitation in the last epoch of Experiment 3 did not reach the significance level, the evidence could increase continuously with more repetitions, and ultimately ‘switching on’ the cueing learning process. Consequently, participants may also show contextual facilitation with more repetition blocks in Experiment 3. This would be an interesting question for future studies. In addition to modify the contextual cueing effect by switching-on (or off) the cueing learning process, the expectations of the upcoming information could also modify contextual cueing effect by changing the response thresholds (i.e., how much information is needed to confirm that the target has been recognized) on the repeated context, hence lead to different amount of contextual cueing effect under different global repetition conditions.

In summary, our findings showed that global repetition of visual stimuli affects (i.e., increase or reduce) contextual learning. This finding suggests that the human visual system may devote more cognitive resources, such as attention and working memory, to process frequently repeated contexts. However, if the repeated context appears infrequently, it may be more efficient to stop context learning during the search to spare cognitive resources.

## Author Contributions

XZ, AZ, LA, LJ, and HL: conception and results interpretation. XZ and AZ: experimental design and data analysis. XZ: data collection. XZ, AZ, LJ, and LA: drafting. AZ, LJ, LA, and HL: revision. LA and HL: final approval. All authors agreed to be accountable for the content of the work.

## Conflict of Interest Statement

The authors declare that the research was conducted in the absence of any commercial or financial relationships that could be construed as a potential conflict of interest.

## Footnotes

1 The ‘expectations of repetitions of the following stimulus’ and the ‘switch on/off process’ could be either explicitly or implicitly according to top-down or bottom-up cues. Previous studies do not agree upon the implicit vs. explicit nature of contextual spatial memory. Although our current study offers some evidence on the explicit nature of contextual cueing, we could not make any strong conclusions because the effect size was very low ($\eta_{p}^{2}$ = 0.006). Therefore, in the current discussion, we choose to not distinguish implicit or explicit mechanisms of expectations and ‘switch on/off processes.
